# The Complicated Nature of Somatic mtDNA Mutations in Aging

**DOI:** 10.3389/fragi.2021.805126

**Published:** 2022-01-10

**Authors:** Monica Sanchez-Contreras, Scott R. Kennedy

**Affiliations:** Department of Laboratory Medicine and Pathology, University of Washington, Seattle, WA, United States

**Keywords:** mtDNA, somatic mutations, aging, mitochondria, mutagenesis, sequencing

## Abstract

Mitochondria are the main source of energy used to maintain cellular homeostasis. This aspect of mitochondrial biology underlies their putative role in age-associated tissue dysfunction. Proper functioning of the electron transport chain (ETC), which is partially encoded by the extra-nuclear mitochondrial genome (mtDNA), is key to maintaining this energy production. The acquisition of *de novo* somatic mutations that interrupt the function of the ETC have long been associated with aging and common diseases of the elderly. Yet, despite over 30 years of study, the exact role(s) mtDNA mutations play in driving aging and its associated pathologies remains under considerable debate. Furthermore, even fundamental aspects of age-related mtDNA mutagenesis, such as when mutations arise during aging, where and how often they occur across tissues, and the specific mechanisms that give rise to them, remain poorly understood. In this review, we address the current understanding of the somatic mtDNA mutations, with an emphasis of when, where, and how these mutations arise during aging. Additionally, we highlight current limitations in our knowledge and critically evaluate the controversies stemming from these limitations. Lastly, we highlight new and emerging technologies that offer potential ways forward in increasing our understanding of somatic mtDNA mutagenesis in the aging process.

## 1 Introduction

Aging is broadly defined as the progressive loss of physiological homeostasis over time and is marked by significant alterations at both the molecular and cellular levels that are associated with an ever-increasing probability of pathology and death. These changes have been broadly categorized into nine types or hallmarks (López-Otín et al., 2013). The relative contribution of these hallmarks to aging and associated pathologies remains under intense debate. While the molecular processes that drive these hallmarks undoubtedly conspire to drive aging, we focus on two hallmarks in this review: mitochondrial dysfunction and genome instability as it relates to mitochondrial DNA (mtDNA).

With only a few noted exceptions, mitochondria are the main source of cellular energy in eukaryotes. These organelles process dietary reducing equivalents and oxygen through the electron transport chain (ETC) to produce ATP via oxidative phosphorylation (OXPHOS). Mitochondria are involved in other important cellular functions such as calcium signaling, iron-sulfur cluster biosynthesis, lipid biosynthesis and apoptosis (Reviewed in (Kowaltowski, 2000)). To varying extents, different cell types rely on these different functions, which, in turn, determines their intracellular localization, dynamics, number, and respiratory flux (Fernández-Vizarra et al., 2011; Herbers et al., 2019). As organisms age, these different mitochondrial processes degrade to differing extents and in tissue specific ways (Cocco et al., 2005; Hebert et al., 2010; Liu, 2013; Kent and Fitzgerald, 2016). A lingering question in the field of aging biology concerns the source of this dysfunction.

As a consequence of an endosymbiotic event ∼2 billion years ago that gave rise to mitochondria, these organelles have retained a small rudimentary genome that, in animals, is comprised of a circular double-stranded DNA molecule present in dozens to thousands of copies per cell (Gray, 2012). The relatively small genome (∼16.5 kbp in humans) is extremely compact and encodes a total of 37 genes: 22 tRNAs, two mitochondrial ribosomal RNAs, and 13 peptides that comprise essential components of the ETC. As such, proper maintenance of the genetic information is essential for energy production and therefore maintaining cell homeostasis. One long-standing hypothesis in aging research is that the loss of genetic information encoded by the mtDNA is an important driver of aging (Cottrell and Turnbull, 2000).

With limited DNA repair capacity and higher replicative index, mtDNA has a substantially higher *de novo* mutation rate compared to nDNA (Reviewed in (Larsson, 2010)). The mitochondrial genome is maternally inherited, with most mtDNA within a cell and organism being an exact copy of the original maternal mtDNA pool, a phenomenon known as homoplasmy. However, mtDNA is susceptible to mutations within the germline, which can result in a number of devastating maternally inherited diseases (Wallace, 1999). In addition to causing overt disease, mtDNA mutations can be present at lower levels, a condition known as heteroplasmy. The heteroplasmic allele fraction can range from very low levels to near homoplasmy and can be inherited or occur *de novo* within somatic tissues during aging and development. Because of the multi-copy nature of mtDNA, it is estimated that the phenotypic threshold for pathogenic heteroplasmies is ∼60–90% of mitochondrial genomes within a cell (Rossignol et al., 1999; Rossignol et al., 2003). To add more complexity to the condition of heteroplasmy, the occurrence and frequency of mtDNA mutations may have different outcomes depending on the timing of their occurrence, the specific tissue in which they arise, and the totaly mtDNA content of the cell (Wallace and Chalkia, 2013; Stewart and Chinnery, 2015; Filograna et al., 2019). Despite decades of study, the complex nature of mitochondrial genetics has made the exact role of somatic mtDNA mutations in aging difficult to discern. In this review, we focus on the complicated observational and experimental evidence suggesting that, at least in some capacity, somatic mtDNA mutations are involved in the aging process with an emphasis of when, where, and how these mutations arise during aging. Additionally, we highlight current limitations in our knowledge and critically evaluate the controversies stemming from these limitations. Lastly, we highlight new and emerging possibilities that offer potential ways forward to increase our understanding of somatic mtDNA in the aging process.

## 2 Correlations Between Aging, Disease, and mtDNA Mutations

A significant body of observational data suggests that the genetic instability of mtDNA in somatic cells is a fundamental phenotype of aging. Numerous studies have shown that mtDNA deletions increase across human tissues during aging (Corral-Debrinski et al., 1992; Cortopassi et al., 1992; Cao et al., 2001; Wanagat et al., 2001; Greaves et al., 2006; Kraytsberg et al., 2006). These deletions tend to be large ( > 1 kb) and lead to a loss of respiratory capacity in the cell (Cao et al., 2001; Wanagat et al., 2001; Kraytsberg et al., 2006). One such deletion in humans, the 4,977bp “common deletion”, is highly prevalent across tissues and has been proposed to be used as a biomarker to discriminate physiological from accelerated aging (Eshaghian et al., 2006; Meissner et al., 2008; Vecoli et al., 2020). Interestingly, a recent in-depth cataloging of deletions in human skeletal muscle using a next-generation sequencing (NGS) method without prior PCR amplification noted a substantial increase in skeletal muscle late in life, but the “common deletion” was not the most abundant species (Lujan et al., 2020). Indeed, studies in other organisms have found a similar age-dependent accumulation of mtDNA deletions (Brossas et al., 1994; Schwarze et al., 1995; Vermulst et al., 2008a). While far more difficult to detect, point mutations have also been noted to increase with age in both humans and mice, suggesting that mtDNA mutations may be a universal component of aging in mammals (Vermulst et al., 2007; Kennedy et al., 2013; Arbeithuber et al., 2020).

Evidence that somatic mtDNA mutations are more than just an epiphenomenon of aging stems from observations that these mutations are frequently further elevated in common diseases of aging and have clear effects on ETC function in the cells that harbor these mutations. For example, neuronal tissues are exquisitely sensitive to mitochondrial dysfunction and mtDNA mutations, both of which increase during aging (Reviewed in (Schon and Manfredi, 2003; Grimm and Eckert, 2017)). Indeed, several common neurodegenerative diseases frequently exhibit an increased burden of somatic mtDNA mutations with associated mitochondrial dysfunction. Several studies have noted highly elevated levels of reactive oxygen species (ROS) and damaged mtDNA bases in Alzheimer’s disease (AD) brains and elevated levels of deletions and point mutations in several brain regions that frequently correlate with the loss of Complex IV activity (Corral-Debrinski et al., 1994; Lin et al., 2002). Studies have also noted the occurrence of specific and recurrent somatic mutations in the mtDNA Control Region in AD patients. The Control Region contains important regulatory regions, such as the heavy-strand origin and transcriptional promoters, and these mutations are associated with reduced mtDNA light-strand transcription and mtDNA copy number in the frontal cortex (Coskun et al., 2004). In addition to AD, mitochondrial dysfunction is a common phenotype observed in brain tissue from Parkinson’s disease (PD) patients. There is a strong association mitochondrial dysfunction and mtDNA mutations in PD that is supported by a variety of observations. (Park et al., 2018). Using Complex IV deficiency as an indicator of mitochondrial dysfunction in individual cells of the SN, Bender *et al.* reported that deficient cells have more somatic mtDNA deletions in PD (Bender et al., 2006). A similar finding has been noted for point mutations, as well (Lin et al., 2012). Mutations in *POLG*, the nuclear encoded mtDNA replicative polymerase, are also associated with familial PD, albeit likely from mtDNA depletion, providing orthogonal evidence supporting a link between mtDNA maintenance and neurodegeneration (Luoma et al., 2007). In addition to neurodegenerative diseases, increased mtDNA mutation loads have also been reported in a number of non-neurodegenerative diseases, including diabetes (Nomiyama et al., 2002), sarcopenia (Herbst et al., 2007; Shah et al., 2009), macular degeneration (Kenney et al., 2010; Atilano et al., 2021), heart disease (Matam et al., 2014), and ulcerative colitus (Baker et al., 2019), suggesting widespread, but tissue specific effects.

Collectively, these studies have provided evidence that the accumulation of somatic mtDNA mutations is a phenotype of aging and a potential causative process in several age-related disease, but questions still remain about the true prevalence of somatic mtDNA mutations in the context on normal aging. This is especially pertinent given the heterogeneous nature of tissue decline during aging. To date, no such detailed survey has been conducted, especially with the newest generation of high accuracy methods (Discussed in Section 6) and this work is lagging behind similar surveys of the nDNA (Moore et al., 2021). While strongly suggestive, these observational studies only provide a correlative association and a direct causal role has been more difficult to establish.

## 3 Experimental Examination of mtDNA Mutations in Aging

**FIGURE 1:**
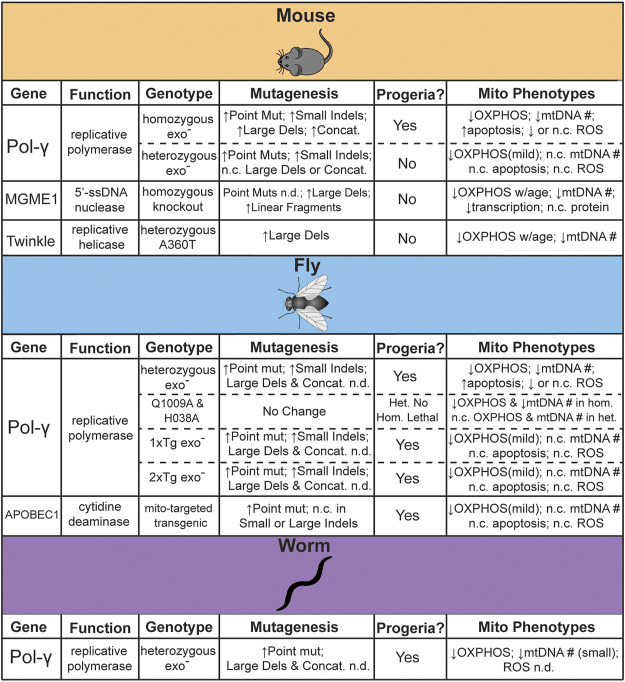
Summary of genetic models reported to test the effects of mtDNA mutagenesis on aging. *n. c. = no change*, *n. d. = not determined*.

Some of the first work attempting to establish the feasibility of a direct link between mtDNA mutations and aging involved the statistical modeling of random genetic drift followed by clonal expansions. These statistical models are consistent with experimental observations at lifespans near that of humans, but they also indicate that mutations were unlikely to accumulate to sufficient levels in short-lived or post-mitotic species (Elson et al., 2001; Kowald and Kirkwood, 2013). Subsequently, a number of genetic models have been engineered with elevated rate of mtDNA mutagenesis in order to directly assess the impact of elevated mtDNA mutagenesis in driving aging (Figure 1). In this section we highlight the animal models that have been generated to test this hypothesis, as well as address the controversies and limitations.

### 3.1 Mouse mtDNA Mutator Models

The first direct evidence for a causative role in aging came from studies in the “mtDNA-mutator” mouse. In this genetic model, mice homozygous for proofreading deficient DNA polymerase *γ* (Pol-*γ* ^exo-^) resulted in a dramatically accelerated aging phenotype across organ systems and a ∼50% reduction in lifespan (Trifunovic et al., 2004; Kujoth et al., 2005). Using a “clone and sequence” approach, both studies noted a ∼4–10-fold increased frequency of mtDNA point mutations with no clear age-associated increase in ROS (Trifunovic et al., 2004; Kujoth et al., 2005). The heterozygous mice did not exhibit these phenotypes and reported no significant difference in mtDNA mutation load, leading to the interpretation that mtDNA mutations drive age-related pathologies (Trifunovic et al., 2004). Interestingly, the high levels of mutations induced by this mouse model exacerbate pathological phenotypes in genetic mouse models of several human diseases. For example, crossing with a mouse model of Alzheimer’s disease (AD) that overexpresses human APP^V717I^, a variant associated with familial AD, led to an increase in A*β*42 and amyloid plaque levels with concomitant brain atrophy (Kukreja et al., 2014). Similarly, loss of Parkin, a component of the mitophagy pathway associated with early-onset familial PD, potentiates the specific degeneration of dopaminergic neurons in the presence of elevated mtDNA mutagenesis and increases motor phenotypes and dopamine metabolism abnormalities (Pickrell et al., 2015). Neither the homozygous Pol-*γ* ^exo-^ nor Parkin knockout strain on their own exhibited any of these phenotypes. Surprisingly, as measured by high accuracy Duplex Sequencing, neither mtDNA mutation frequency nor spectrum changed with the loss of Parkin, suggesting that mitophagy is not acting to remove pathogenic mutations as originally hypothesized (Suen et al., 2010).

While this mouse model has shown that high aggregate loads of somatic mtDNA mutations can affect cell physiology and homeostasis, its relevance to aging has been controversial. The interpretation that high mutation levels drive aging was based on the observation that the homozygous Pol-*γ* ^exo-^ mouse appeared to have a much higher mutation frequency than the heterozygous Pol-*γ* ^exo-^ strain. This finding was challenged when both genotypes were assayed for random mutations with the Random Mutation Capture (RMC) method, which has a significantly reduced background compared to “clone and sequence” approaches (Bielas and Loeb, 2005; Vermulst et al., 2008a). Surprisingly, the frequency of mtDNA point mutations were nearly identical between the heterozygous and homozygous mice, with both being > 100-fold higher than wild-type age-matched controls (Vermulst et al., 2007). Instead, using a modified form of RMC, large deletions (*i.e.*, > 1 kb) were substantially higher in the homozygous mouse compared to the heterozygous one, which was not increased relative to wild-type controls (Vermulst et al., 2008b). These observations, along with the absence of reduced lifespan in the heterozygous strain, led to the conclusion that deletions, not point mutations, act as the driver of age-associated pathologies. These findings have widely been interpreted as meaning that mtDNA point mutations have no significant role in aging or its pathologies. However, several lines of evidence suggest that the relative contributions of deletions and point mutations to physiological decline is more complicated than widely accepted.

A prediction of the mtDNA deletion hypothesis is that the loss of large segments of mtDNA would result in significant disruption to mitochondrially encoded ETC components due to the loss of both tRNA and mRNA transcripts. A molecular characterization of mitochondria from homozygous Pol-*γ* ^exo-^ mice noted no large changes in mitochondrial tRNA and mRNA transcripts or overall *in organello* protein translation rates, as expected if deletions were not significantly disrupting mtDNA function. Furthermore, the analysis of ETC supercomplex assembly and subunit turnover suggested that the ETC was unstable. Given that the ETC components were efficiently synthesized, which would be affected by deletions, but were unstable and rapidly turned over, which would be affected by deleterious point mutations, these data were interpreted as suggesting that deletions were not driving ETC dysfunction (Edgar et al., 2009). However, it has been noted that the reliance of this study on bulk tissue analysis would obfuscate the contribution of randomly generated deletions, which, individually, would be quite rare and only affect a small number of related cells, but could result in a high aggregate burden within a tissue (Discussed further in Section 6) (Vermulst et al., 2009). In an unrelated study, detailed analysis of the heterozygous Pol-*γ* ^exo-^ mice, which have a mutator phenotype without an attenuated lifespan, have documented a number of accelerated age-related phenotypes, including significant declines of neuromuscular and locomotor activity, O_2_ consumption, heat production, and ETC activity (Dai et al., 2013; Fuke et al., 2014). This highlights the impact of somatic mtDNA point mutations on healthspan that is independent of lifespan. More recently, mice lacking MGME1, a single-stranded DNA exonuclease that processes 5′-mtDNA ends generated during mtDNA replication, accumulate linearized and deletion-harboring mtDNA molecules at a similar level as the homozygous Pol-*γ* ^exo-^ mouse, as determined by long-extension PCR, but does not show a premature aging phenotype (Matic et al., 2018). Similarly, a knock-in mouse of a dominant negative A360T mutation in *Twinkle*, the mtDNA replicative helicase, accumulates a significant burden of mtDNA deletions, as determined by long-extension PCR, without significantly altering lifespan. Interestingly, this genetic model exhibited an increased incidence of COX-deficient muscle fibers in old age (18–24 months), indicating mitochondrial dysfunction, but with no apparent effect on physical performance (Tyynismaa et al., 2005). Additional work, especially with more quantitative methods, is needed to better understand the differing effects on aging between these different mouse models.

### 3.2 Invertebrate mtDNA Mutator Models

Dissecting the importance of somatic mtDNA mutations in invertebrates has proven to be as equally complicated, if for differing reasons. As originally noted in studies statistically modeling the accumulation of mutations during aging, short-lived and post-mitotic organisms are unlikely to generate and clonally expand mutations to a level that is expected to interfere with tissue function. Consistent with this hypothesis, a *D. melanogaster* heterozygous mtDNA-mutator model harboring the equivalent Pol-*γ* ^exo-^ mutation as the mouse model exhibited no decrease in lifespan, even when propagated through the maternal line for five generations (Kauppila et al., 2018a). Using a PCR and sequence strategy this model exhibited a strong mutator phenotype, consistent with the elevated levels seen in the mouse model. We have independently confirmed this strong mutator phenotype by high accuracy sequencing (Andreazza et al., 2019). Surprisingly, only when exonuclease deficiency was maintained as a compound heterozygote with a catalytically deficient Pol-*γ* allele for > 35 generations did mutations rise to a level sufficient to reduce lifespan and exhibit metabolic dysfunction. More recently, we generated and characterized an analogous transgenic *D. melanogaster* model expressing one or two copies of Pol-*γ* ^exo-^ (Samstag et al., 2018). This model was engineered such that the transgene was under the control of the endogenous *PolG* promoter and *cis*-regulatory elements in order to avoid mtDNA depletion resulting from *PolG* overexpression (Martínez-Azorín et al., 2008). In contrast to the original model, this transgenic model showed a Pol-*γ* ^exo-^ dose-dependent increase in mutation load, determined by high accuracy sequencing, that correlated with premature aging-like phenotypes (*i.e.*, reducing climbing capacity, neurodegeneration, increased mitochondrial dysfunction, and reduced lifespan). Given that this model exhibited no apparent effect on mtDNA copy number or *PolG* expression level, the reason for the discrepancy in lifespan and aging phenotypes between the two models is unclear.

In addition to *D. melanogaster*, a heterozygous *C. elegans* Pol-*γ* ^exo-^ mutator model has also been developed (Haroon et al., 2018). Although originally created to facilitate the rapid screening of genetic and cellular pathways that modify mtDNA disease phenotypes, this worm strain recapitulates several phenotypes seen in both the *D. melanogaster* and mouse Pol-*γ* ^exo-^ mutator models. These include a ∼70-fold increase in mtDNA mutations (determined by RMC), an elevation in mitochondrial dysfunction, and shortened lifespan (Haroon et al., 2018). Interestingly, manipulation of several genetic pathways important in aging, including IGF-1/insulin signaling, mitophagy, and the mitochondrial unfolded protein response, were shown to ameliorate the negative phenotypes associated with elevated mtDNA mutation loads, suggesting an intimate, albeit more indirect, interaction between mtDNA mutagenesis and aging.

In addition to the predominant Pol-*γ* ^exo-^ model, an alternative mtDNA mutator model has been developed in *D. melanogaster* that transgenically expresses mitochondrially targeted APOBEC1 (mtAPOBEC1) (Andreazza et al., 2019). APOBEC1 (Apolipoprotein B (apoB) mRNA editing catalytic polypeptide 1) catalyzes the deamination of cytidine and, in the absence of its RNA-binding accessory subunit, specifically acts on DNA and leading exclusively to C → T/G → A mutations (Harris et al., 2002). In contrast to the original Pol-*γ* ^exo-^ fly model that exhibited no significant aging phenotypes, mtAPOBEC1 severely limited mitochondrial function, organismal vitality, and lifespan. Interestingly, the overall mutation frequencies and heteroplasmic load between the two fly models were comparable. Importantly, owing to its mode of action as a base editor, mtAPOBEC1 exclusively induced G → A/C → T mutations. Interestingly, mutations induced by mtAPOBEC1 where significantly more likely to be nonsynonymous and had a much higher predicted pathogenicity score, leading to the conclusion that the functional impact of a mutation was more important than the load of mutations in affecting organismal fitness (Andreazza et al., 2019). Taken together with the *C. elegans* and transgenic *D. melanogaster* Pol-*γ* ^exo-^ models, three of the four available invertebrate mtDNA mutator models suggest that mtDNA mutations can, in fact, limit the lifespan of short-lived post-mitotic species.

### 3.3 Limitations of mtDNA Mutator Models

An issue with the Pol-*γ* mutator models in both vertebrates and invertebrates is that the loss of exonuclease activity affects polymerase processivity and likely interferes with the final processing steps of replication (Bailey et al., 2009; Szczepanowska and Foury, 2010; Macao et al., 2015; Nissanka et al., 2018). Consequently, Pol-*γ* mutator models frequently exhibit significant reduction in mtDNA copy number, especially in the homozygous state. The MGME1 and Twinkle mtDNA “deleter” mouse models similarly suffer from this issue. mtDNA copy number is strictly regulated by the cell, with the loss of mtDNA linked to a metabolic dysfunction and a number of age related diseases (Reviewed in (Castellani et al., 2020)). Moreover, the loss of mtDNA may be especially important in the context of heteroplasmy, as the reduced copy number may allow mutations normally below the phenotypic threshold to rise above this level and induce dysfunction. Ultimately, the inability to clearly distinguish between the effects of mtDNA copy number and enhanced mutagenesis complicates the interpretation of the role of mtDNA mutations in aging across all the Pol-*γ* models. The mtAPOBEC fly model, described above, was developed, in part, to address this issue and does not exhibit altered mtDNA copy number or the generation of insertion or deletion mutations. Unfortunately, this animal model is currently only available in flies, so its relevance to mammalian aging remains unexplored, but may be worth pursuing given its high specificity for point mutations.

Another key limitation in the efforts to link aging and somatic mtDNA mutations is that all of the above-described animal models increase the mutation load. While informative for understanding if these mutations are able to drive aging-like phenotypes at high levels, these animal models do not directly address if somatic mtDNA mutations are important in the normal aging process. If mutations do play a role in aging, a prediction would be that a reduction in mutagenesis would lead to an amelioration of age-related pathologies and/or extension of lifespan. To date, only limited attempts have been reported with none focused on lowering mtDNA deletions. Specifically, two fly strains have been engineered to express Pol-*γ* with single amino acid changes (either Q1009A or H1038A) corresponding to highly conserved amino acids that confer an anti-mutator phenotype in evolutionarily related *E. coli* DNA polymerase I (Minnick et al., 1999; Bratic et al., 2015). Both of these alleles exhibited developmental lethality when in a homozygous or hemizygous null state and no alterations in lifespan when heterozygous with the wild-type allele (Kauppila et al., 2018a). Notably, no change in mutation frequency relative to wild-type control was detected in either of these strains.

Importantly for experiments that aim to reduce mtDNA mutagenesis, the balance between exonuclease and polymerase activity is a critical aspect that governs fidelity in all proofreading polymerases, with anti-mutators variants frequently slowing the rate of polymerization and allowing more time for removal of misincorporations by the proofreading exonuclease domain (reviewed in (Bebenek and Ziuzia-Graczyk, 2018)). Biochemical analysis of the equivalent amino acid changes in human Pol-*γ* exhibited enhanced exonuclease activity, consistent with potential anti-mutator action, but poor processivity and synthesis rates that resulted in a significant reduction in mtDNA copy number *in vivo* in the homozygous and hemizygous strains. These results indicate that the Pol-*γ* ^Q1009A^ and Pol-*γ* ^H1038A^ variants likely act as a functional null and further suggest that the heterozygous strains are functionally acting as hemizygous wild-type, which would not be expected to alter mutation frequency without altering mtDNA copy number (Bratic et al., 2015; Samstag et al., 2018).

The generation of a large library of randomly generated mutants in *E. coli* DNA polymerase I demonstrated a number of mutants that increased fidelity of base selectivity by > 10-fold without significantly altering polymerase activity, indicating that better variants to test the impact of lower mutagenesis may exists (Loh et al., 2007). Indeed, work in the yeast Pol-*γ* homolog, Mip1, identified A256T (A300 in human Pol-*γ*) as a moderate anti-mutator without any apparent loss in polymerase activity, suggesting that such screening would likely be fruitful in identifying other variants with substantially reduced mutation rates that could be used to directly test if lowering mtDNA mutagenesis extends lifespan (Foury and Szczepanowska, 2011).

Collectively, the genetic models that have been generated generally indicate that elevated mtDNA mutations are able induce aging-like phenotypes in both vertebrate and invertebrate models. However, there is a surprising amount of phenotypic variability across these models, suggesting that the influence of mtDNA in aging is more nuanced and complicated than originally assumed. Some of the variability and conflicting data likely comes down to specific aspects of these models, including the relative generation of point mutation versus deletions, the specific genome engineering and crossing schemes used to generate the models, and the technology used to evaluate mutation loads. Each of these specifics can significantly influence the interpretation of results. As such, given what we now know about these animal models, as well as mtDNA biology, it will be important in future experiments to, where possible, carefully control for these possibilities and provide complete experimental procedures.

## 4 Clonal Expansions in Aging

An aspect that is frequently overlooked in studies focused on mtDNA mutations and aging is the role of clonal expansions (also referred to as clonal mosaicism). This phenomenon refers to genetic heterogeneity that arises from post-zygotic mutations that propagates within tissues over time due to cell division and tissue remodeling. While the biology underlying this phenomenon is poorly understood, specific mutations are frequently observed to accumulate to high levels within individual cells and tissues and are frequently associated with defects in OXPHOS. These mosaic fields have been observed in numerous different tissue types in both aging and disease and appear to have some level of tissue specificity. Phenotypically, clonal fields are largely characterized by patches of tissues, some quite large, exhibiting a loss in OXPHOS activity. For example, colonic epithelium has shown that normal aging in associated with an increase in clonal fields lacking OXPHOS activity that are the result of mtDNA mutations (Taylor et al., 2003; Greaves et al., 2014). Similar findings have been observed in stomach, prostate, and small intestines of normally aged individuals, as well as blood of Huntington’s disease patients (Fellous et al., 2009; Greaves et al., 2010; Wang et al., 2021).

The timing of occurrence and functional relevance of the mutation driven OXPHOS mosaicism in aging is poorly understood. A long-standing hypothesis has proposed that the mtDNA mutations detected in the somatic tissue are the result of low-level mtDNA mutations acquired during embryogenesis or early in life that expand over time and not the result of late arising *de novo* events (Elson et al., 2001). Observational and mathematical modeling studies support this hypothesis. Using a combination of conventional NGS and high sensitivity RMC to study the burden of clonal expansions and *de novo* mtDNA mutations, respectively, in human colonic crypts, Greaves *et al.* noted a significant expansion of clones without an associated increase in *de novo* mutations during aging, supporting the hypothesis that age-associated OXPHOS mosaicism arises from early life events, at least in highly proliferative tissues (Greaves et al., 2014). Interestingly, a similar pattern has been seen in non-proliferative tissues. In human cerebellum, relatively high heteroplasmies ( > 10%) have been found to be prevalent, but do not appear to change with age, suggesting that the observed age-related mtDNA mutation reflects either *de novo* mutations acquired in early development or low-level germline mutations that clonally expanded (Wei et al., 2017). In a separate study in human brain, the aggregate load of large ( > 1 kb) mtDNA deletions increased with age in human brain, but the diversity of deletion breakpoints did not increase, suggesting that the total load of mtDNA harboring deletions is derived from relatively few deletion events (Taylor et al., 2014). Lastly, mathematical modeling, as well as the spatial distribution and clustering of somatic mtDNA mutations, further supports that most age-associated accumulated mtDNA mutations are the result of clonal expansion of pre-existing mutations (Elson et al., 2001; Khrapko et al., 2004). Taken together, these data suggest that clonal expansions of mutations that result in loss of OXPHOS activity is a major aspect of aging. However, interpretation that these mutations are derived from early events has been controversial, as it has been noted that somatic mtDNA mutagenesis could accelerate in late life with different rates of clonal expansion (Popadin et al., 2014).

### 4.1 Testing the Importance of Clonal Expansions in Aging

While these observational data suggest that early arising mutations are the likely source of age-related OXPHOS decline, only a few studies have been performed to experimentally test their contribution to aging. For example, recent work in an intestinal tumor mouse model indicates that when combined with enhanced mtDNA mutagenesis, a growth advantage is conferred by a remodeling of the metabolic network (Smith et al., 2020). Unfortunately, in many of the previously developed experimental animal models, highlighted in Section 3, the heteroplasmic level (or variant allele fraction), is often only descriptively alluded to by the presence of OXPHOS negative cells and is not directly reported or is unable to be rigorously quantified due to the method(s) used. Thus, it is unclear what role, if any, clonal expansions play in driving the observed phenotypes in these experimental animal models. A prime example concerns the Pol-*γ* ^exo-^ mouse. The generation of homozygous Pol-*γ* ^exo-^ mice necessarily requires the transmission of mutated mtDNA molecules through the germline from a heterozygous Pol-*γ* ^exo-^ female. The result is that a significant burden of mutations is inherited and will clonally expand to high levels during development. Indeed, in the initial characterization of this mouse model, loss of OXPHOS activity was noted in histological examination of tissues compared to wild-type controls. OXPHOS mosaicism in heterozygous Pol-*γ* ^exo-^ mice was also noted to be present, albeit at a reduced levels (Vermulst et al., 2008b). Importantly for these early studies, crossing information regarding which parent the Pol-*γ* ^exo-^ allele came from in the experimental mice was not noted. Due to the maternal inheritance of mtDNA, such information is critical in interpreting experiment results in mtDNA mutator animal models. Indeed, a follow up study involving clearly defined crossing schemes designed to evaluate the relative importance of inherited versus somatic mutations in the induction of aging phenotypes has been performed (Ross et al., 2013). Surprisingly, passage of mtDNA mutations arising from a single-generation transient mutational burst in the female germline led to decreased fitness and lifespan in the absence of the Pol-*γ* ^exo-^ allele (Ross et al., 2013; Ross et al., 2015). The introduction of additional somatic mutations exacerbated this phenotype indicating that somatic *de novo* mutations can affect aging in the presence of inherited mutations, but also complicating the interpretations about the role of purely somatic mutagenesis in the initial literature analyzing these animal models.

## 5 Replication or Deoxyribonucleic Acid Damage: What Are the Drivers of Mutagenesis in mtDNA

While mutations in mtDNA are linked to age-related human pathologies, identifying the source of these mutations has also proved complicated and remains under considerable debate (Sevini et al., 2014; Szczepanowska and Trifunovic, 2017; Chocron et al., 2019). Understanding the source of these mutations can provide clues to physiological and biochemical processes that may drive aging, even in the absence of any direct effect. Broadly speaking, mutations in the mtDNA almost exclusively occur as either single nucleotide point mutations or deletions that range in size from a single base to many kilobases. Few insertions, especially large ones, are observed (Kennedy et al., 2013; Lujan et al., 2020). Point mutations are thought to occur by base misincorporation events due to either unrepaired DNA damage or base-selectivity errors by DNA polymerase Pol-*γ*.

### 5.1 The Surprising Lack of Reactive Oxygen Species Linked Mutations

The most frequently cited source of age-associated point mutations stems from the long hypothesized free radical theory of aging (and associated mitochondrial free radical theory of aging), which proposes that aging is the result of accumulating molecular damage caused by free radical species such as ROS that are a normal by-product of cellular metabolism (Harman, 1956; Harman, 1972). Central to this theory is that ROS produced by the mitochondria are in close proximity of mtDNA, making it susceptible to oxidative damage and mutation and resulting in a feedback loop of ever-increasing ROS production and mutation (Harman, 1972; Miquel, 1992; Kadenbach et al., 1995). In addition to its proximity to the ETC, mtDNA is more susceptible to damage due to its more limited protective and repair systems compared to the nDNA (Mandavilli et al., 2002). However, the connection between mtDNA oxidative damage, mtDNA mutagenesis, and aging appears to be more complicated than anticipated.

Oxidation of deoxyguanosine to 8-oxo-7,8-dihydroguanine (8-oxo-dG) lesion in mtDNA has been found to accompany normal aging in multiple tissues (Mecocci et al., 1993; Short et al., 2005; Ma et al., 2016). The presence of 8-oxo-dG is mutagenic by its preference to pair with deoxyadenosine, which ultimately results in G → T transversions (Cheng et al., 1992). While studies have shown that 8-oxo-dG increases with age in mtDNA, we and others have shown that, contrary to expectations, the frequency of somatic mtDNA G → T/C → A transversions is substantially lower than G → A/C → T and T → C/A → G transitions in human brain and does not increase with age (Zheng et al., 2006; Kennedy et al., 2013; Williams et al., 2013). More recent work in mice and flies have noted a similar bias, suggesting evolutionary conservation of the mutagenic processes active in mitochondria (Itsara et al., 2014; Samstag et al., 2018; Arbeithuber et al., 2020; Sanchez-Contreras et al., 2021). Lastly, the lack of G → T/C → A transversions and a preponderance of G → A/C → T and T → C/A → G transitions is also reflected in population level SNPs, suggesting that 8-oxo-dG is not a source of mutations in the germline at levels high enough to drive genetic variation even over long periods of time (Belle et al., 2005; Kennedy et al., 2013). Indeed, recent analysis of mtDNA mutations in individual mouse oocytes using ultra-high accuracy sequencing supports this conclusion (Arbeithuber et al., 2020).

Mitochondria have several antioxidant defenses thought to prevent or repair oxidative damage to their genome. Several glycosylases with overlapping and complementary activities are active on mtDNA to remove ROS-induced damage to deoxyguanosine (Prakash and Doublié, 2015). Several studies have evaluated the importance of mitochondrial base excision repair (BER) to mtDNA maintenance. Knockout of mitochondrial OGG1 and NEIL1, which remove 8-oxo-dG and formamidopyrimidine lesions, respectively, in mice results in mitochondrial dysfunction and a metabolic syndrome phenotype (Vartanian et al., 2006; Sampath et al., 2012). Conversely, overexpression of the mitochondrial isoform of OGG1 is protective against the deleterious effects of a high-fat, high-sugar diet, which is known to result in increased ROS production (Matsuzawa-Nagata et al., 2008; Komakula et al., 2018). Together, these studies suggest that BER plays an important role in mtDNA integrity. However, characterization of mtDNA mutations was not performed in these studies. Interestingly, in a different study using unrelated mouse lines, knockout of the mitochondrial form of OGG1 and MUTYH, which removes deoxyadenosine mispaired with 8-oxo-dG, resulted in no increase in mtDNA mutations in old mice (∼24 months), nor did mice lacking SOD2 in the heart on its own or when combined with the loss of mitochondrial OGG1. Furthermore, mice deficient in OGG1 and MUTYH and bred for five generations resulted in no increase in inherited mtDNA mutations in the maternal lineage (Kauppila et al., 2018b). These data were interpreted to mean that BER, despite its presence in the mitochondria, does not act to prevent mutagenesis of mtDNA.

While strongly suggestive that ROS is not the major driver of mtDNA mutagenesis, some caution is warranted before fully exonerating ROS in mtDNA mutagenesis. First, FapyG (2,6-diamino-4-hydroxy-5-formamidopyrimidine) is reported to be produced more efficiently than 8-oxo-dG *in vivo*, which may be the result of lower oxygen concentrations and the presence of reducing equivalents such as thiols in the cellular environments (Pouget et al., 2002). Therefore, the loss of repair may only modestly affect the mutation load of mtDNA. Second, the frequency of somatic G → T/C → A mutations in wild-type mouse heart is reported to be on the order of 10^–7^, approximately two-orders of magnitude below the reported frequencies in wild-type controls in this study, suggesting technical limitations in the approach used to detect the mutations (Arbeithuber et al., 2020). Third, ROS-induced damage to deoxyguanosine may not be the primary mode of oxidative damage to DNA. Indeed, 5-hydroxypyrimidines, a dominant product of hydroxyl radical chemistry strongly induce C → T/G → A transitions (Feig et al., 1994; Purmal et al., 1994; Gates, 2009). Consistent with this possibility, Vermulst et al. noted that mice overexpressing mitochondrially targeted catalase (MCAT), which reduces hydroxyl radical levels, exhibit a lower overall mutation frequency largely driven by fewer C → T/G → A transitions (Vermulst et al., 2007). Examination of the effects of hydrogen peroxide on ssDNA in yeast has also highlighted a specific mutational signature that consistent with this observation (Degtyareva et al., 2019). Lastly, biochemical analysis of Pol-*γ* has demonstrated translesion-like activity that correctly incorporates deoxycytidine across from 8-oxo-dG, so even in the absence of antioxidant and repair mechanisms, 8-oxo-dG induced mutations may be actively prevented by the polymerase itself and may explain the disconnect between the high prevalence of 8-oxo-dG in mtDNA and the lack of associated mutations (Graziewicz et al., 2007; Cline, 2012). Taken together, the available evidence suggests rejecting a role of ROS in mtDNA integrity may be premature, but does support the notion that 8-oxo-dG is not the primary mutagenic lesion in mtDNA.

### 5.2 Alternative Drivers of mtDNA Mutagenesis

If 8-oxo-dG is not the major mutagenic driver, then what is? The lack of canonical ROS-induced mutations has led to the proposal that either spontaneous deaminations or base-selectivity errors by Pol-*γ* are the primary driver of point mutations seen during aging (Ameur et al., 2011; Kennedy et al., 2013). Few experiments have been performed to directly test these hypotheses or distinguish between these processes *in vivo*, but several reports have made observations that support one or both possibilities. Early in the discovery and *in vitro* characterization of the eukaryotic polymerases, it was noted that Pol-*γ* easily extends past deoxyuracil and 5-hydroxycytidine and also showed a strong preference for dC:dA mispairs, which subsequently result in a G → A/C → T mutations (Kunkel and Alexander, 1986; Lee and Johnson, 2006). *In vitro* assays involving the copying of DNA by Pol-*γ* show predominantly G → A/C → T mutations, even when controlling for potential confounders such as heat-induced template deamination (Longley et al., 2001; Zheng et al., 2006). Direct oxidation of Pol-*γ* itself is also reported to result in ∼20-fold reduction in exonuclease-mediated proofreading, the loss of which increases transition mutations, suggesting a possible indirect mode of action for ROS in mtDNA mutagenesis during replication (Anderson et al., 2020).

Suggestively, we characterized the distribution of ∼35,000 somatic mtDNA mutations in mouse mtDNA and noted a strong discontinuous strand-asymmetric gradient in the frequency of transitions along the mtDNA that is delimited by the light-strand origin (Ori_L_) and the control region (CR) (Sanchez-Contreras et al., 2021). This pattern is interpreted as being most consistent with an asynchronous strand-displacement replication model proposed by David Clayton and colleagues (Clayton, 1982; Clayton, 2003; Brown et al., 2005). This replication model posits a long-lived single-stranded replication intermediate between the Ori_L_ and CR that is more prone to deamination. The slope of this gradient increases in the major arc during aging and is lost in the absence of Pol-*γ* proofreading activity. These patterns suggest that most point mutations arise from deamination of cytidine or adenosine located in a single-stranded replication intermediate that is a consequence of the asymmetric strand-displacement mechanism. It remains to be seen if deaminations are spontaneous or driven by exposure to ROS.

Much like the experimental work aimed at teasing out the role of mtDNA mutations in driving aging, the molecular source of endogenous mutations has proved as equally enigmatic. Results from several different methods have firmly established that the main source of mutation is unlikely to be 8-oxo-dG, contrary to the long standing free radical theory of aging. Instead, focus has shifted to either base misincorporation errors by Pol-*γ* or spontaneous deamination of cytidine and adenosine. However, the possibility remains that ROS may be a driver of deamination itself or an indirect mutagen arising from damage to Pol-*γ*. One aspect that has received only minor attention is the interplay between DNA damage, repair pathways, and replication and that no one mechanism is the driver of mutagenesis. Experiments that attempt to integrate these different aspects of mtDNA biology are likely to be informative in teasing out the main source(s) of mutations.

## 6 Providing Clarity to the Role of Somatic mtDNA Mutations in Aging With Emerging Technologies

The invention of PCR, along with its continued refinement as a key technology, has steadily increased the ability to detect rare mutations down to the level of individual cells or even individual mtDNA molecules (Kraytsberg et al., 2009; Payne et al., 2015). While effective, most PCR methods are low throughput or require prior knowledge of where in the genome to assay for mutations. The recent development of droplet-digital PCR (ddPCR) has significantly increased the throughput of PCR-based assays (Hindson et al., 2011). The technology makes use of a water-oil emulsion to create tens of thousands of nanoliter-sized PCR reaction “vessels”. This technology has been combined with enzymatic selection methods to remove non-mutated genomes, allowing for the ability to detect both point mutations and deletions in mtDNA (Taylor et al., 2014; Soltys et al., 2019). Similar approaches have been developed that incorporate the use of TaqMan method and multiplexing with different fluorescent mutation-specific probes, allows for the quantification of heteroplasmy in individual cells with reasonable throughput (Maeda et al., 2020). PCR-based assays have the advantage of being relatively inexpensive to perform. However, throughput, while significantly improved, is still limited to a few thousand individual cells/genomes which makes the detection of low frequency heteroplasmies or clones, which are often present at variant allele fractions of < 1 × 10^−3^, difficult. Another significant limitation is that PCR assays must be optimized for each new genome or target area.

The advent of NGS allows for the digital tabulation of many individual DNA fragments in parallel, offering the unique ability to detect low level nucleic acid species within heterogeneous mixtures. This has led to routine sequencing of the entire mtDNA molecule with more accuracy and dramatically increased throughput, resulting in a significant increase in our understanding of mitochondrial genetics in disease (Macken et al., 2021). Early deployment of conventional NGS methods in studying mtDNA in the context aging noted modest changes in point mutations and deletions in humans (Williams et al., 2013), but no significant age-associated changes in other organisms such as wild-type mice (Ameur et al., 2011; Ma et al., 2018), leading to the conclusion that the importance of somatic mtDNA mutations in aging may be organism specific. However, a major limitation to standard NGS platforms is their technical error rate, which ranges from ∼0.1–10% false base calls, depending on the technology, with a practical limit of 0.5–1% for the most commonly used Illumina platform (Glenn, 2011). Several approaches have been developed to increase the accuracy of modern sequencing platforms (Salk et al., 2018). The most widely used approach to reduce this background is consensus-based error correction. This approach compares sequencing information of PCR copies derived from an originating DNA molecule to form a consensus. Reads are determined to be related by the use of uniquely identifying information encoded in the originating DNA, frequently termed “molecular barcodes”, “unique molecular identifiers”, or “single molecule identifiers”, that is propagated to all daughter molecules during PCR. A number of different methods based on this approach have been reported (Reviewed in Salk et al. (2018)), but the most accurate to date is Duplex Sequencing (Schmitt et al., 2012). This approach makes use of double-stranded molecular barcodes that enables the comparison of single-strand consensus information from both strands of a parental DNA fragment and has a reported error rate of ∼2 × 10^−8^ (Abascal et al., 2021). This approach has been used extensively to study somatic mtDNA mutations in a variety of contexts (Kennedy et al., 2013; Pickrell et al., 2015; Hoekstra et al., 2016; Samstag et al., 2018; Andreazza et al., 2019; Arbeithuber et al., 2020; Wu et al., 2020; Broz et al., 2021; Sanchez-Contreras et al., 2021).

**FIGURE 2:**
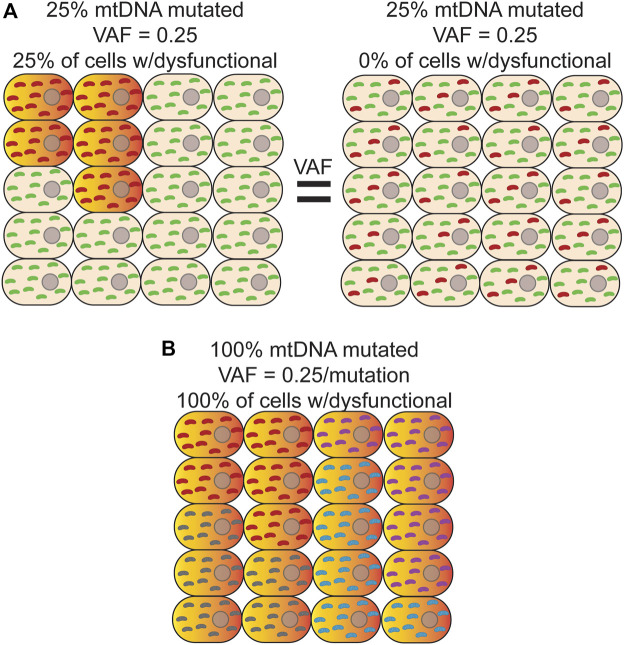
Bulk analysis of mtDNA mutations is unable to distinguish different scenarios with different physiological consequences. **(A)** Bulk sequencing of cells where *left*-25% of cells harbor a homoplasmic mutation (*red*) that causes OXPHOS dysfunction (*orange/red-gradient*) and the remainder contained only wild-type mtDNA (*green*) would have the same variant allele fraction (VAF) as bulk sequencing of a sample where *Right*-100% of cells contain a mutation that is, on average, at 25% heteroplasmy. In both cases, the apparent VAF would be 0.25, but the effect on tissue function would be different. Distinguishing between these two scenarios is only possible with single cell sequencing. **(B)** In this example, every cell is a clonal expansion harboring one of five possible homoplasmic mtDNA mutations (*red, purple, grey, and cyan*) that results in OXPHOS dysfunction. In bulk sequencing, no mutation would be seen to have a VAF > 0.25, which is below the apparent phenotypic threshold.

NGS is typically performed on bulk DNA from thousands to millions of cells, which necessarily decouples variant phasing information between any two reads, resulting in a loss of important biological information such as if a variant is homoplasmic or heteroplasmic within any given cell. This limitation is important given the phenotypic threshold effect imparted by different heteroplasmic levels (Figure 2A) (Wallace and Chalkia, 2013). In addition, phasing a variant to a specific cell allows for potential cell-type assignment and ‘multi-omic’ analysis of transcriptional and epigenetic consequences of a variant that would not otherwise be possible. While still a fairly new technology, sequencing at single-cell resolution has brought about the ability to identify rare cell types (Cao et al., 2017; Rosenberg et al., 2018), better estimates for tissue and tumor heterogeneity (Navin et al., 2011; Zong et al., 2012; Wang et al., 2014), and track developmental lineages (Trapnell et al., 2014; Cusanovich et al., 2015; Athanasiadis et al., 2017; Preissl et al., 2018). Original single-cell approaches used micromanipulation of individual cells into independent reaction wells followed by whole-genome amplification, but suffers from severe scalability problems, as well as the introduction of significant biases arising from sample dropout and nucleic acid amplification (Reviewed in (Navin, 2015)). In response to the need for more high-throughput methods, microfluidic sorting of cells (Rinke et al., 2014), nano-well (Gierahn et al., 2017), emulsion droplet partitioning technologies (Klein et al., 2015; Macosko et al., 2015; Rotem et al., 2015), and combinatorial cellular indexing (Cao et al., 2017; Vitak et al., 2017; Rosenberg et al., 2018) have been successfully developed and deployed to perform whole genome, epigenome, and transcriptome analysis on many thousands of cells.

The deployment of these technologies to study *de novo* mtDNA mutations has, so far, been limited. Due to the much higher mutation rate of mtDNA, most applications have used *de novo* mutations as a type of barcode to perform cell lineage tracing (Xu et al., 2019; Lareau et al., 2021; Ludwig et al., 2019). As noted above, bulk analysis of DNA results is an average of the heteroplasmic state of all cells in the sample, obscuring the true heteroplasmic state of each constituent cell. Thus, this approach is unable to distinguish between scenarios in which a small population of cells is homoplasmic for a variant versus 100% of cells harboring the same variant as a lower heteroplasmic level (Figures 2A,B). As a byproduct of using single-cell technologies to construct cell lineages with mtDNA variants, these initial studies suggest that both scenarios are present in cell populations (Ludwig et al., 2019; Xu et al., 2019). Moreover, heteroplasmic mutations can vary considerably in their allele fraction between cells but appear to be stably transmitted at or near the same level during the majority of cell divisions, suggesting that significant changes in heteroplasmy is an infrequent event, at least in human hematopoietic stem and progenitor cells (Ludwig et al., 2019). Interestingly, combining variant information with chromatin accessibility data within the same cell indicates that when mtDNA mutations are likely pathogenic and rise above the phenotypic threshold, they are able to impart changes in the epigenome and can have important consequences for cell fate decisions during hematopoietic differentiation (Lareau et al., 2021). Applying these approaches explicitly in the context of aging will likely be highly fruitful in better understanding the cellular and physiological impact of somatic mtDNA mutations.

To date, most single-cell approaches involve the dissociation of cells, which results in the loss of spatial information regarding how mtDNA mutations are distributed within a tissue. The lack of this information is likely an important aspect that hinders the ability to interpret their potential physiological impact. Spatial genomics is a rapidly emerging field of research that aims to layer spatial information to sequencing data at or near single-cell resolution. Current technologies have largely focused on obtaining the distribution of transcripts within tissues, which, much like single-cell approaches, could be harnessed to obtain spatial distribution of mtDNA heteroplasmies. To date, two main approaches have been developed that are likely amenable to the study of mtDNA mutations *in situ* with only minor modifications: spatially-aware RNA-Seq (Fazal et al., 2019; Rodriques et al., 2019; Srivatsan et al., 2021; Stickels et al., 2021), and direct *in situ* sequencing (Ke et al., 2013; Lee et al., 2014; Gyllborg et al., 2020). To date, neither of these approaches have been explicitly applied to detecting mtDNA variants. However, mitochondrial transcripts, which reflect the underlying mtDNA sequence, are highly abundant in transcriptomic data and are typically explicitly ignored in most studies. These exciting emerging technologies potentially open up the possibility to study low level mtDNA heteroplasmies within the context of intact tissue with the potential ability to correlate this information with histological characterization of ETC function in a similar fashion to what has been done previously with laser microdissection (Greaves et al., 2014).

## 7 Concluding Remarks

The accumulation of somatic mtDNA mutations over time is undoubtedly a phenotype of aging. However, establishing the physiological impact of these mutations, especially as it pertains to aging, has proved difficult. A number of animal models have been developed with the intention of directly testing the impact of elevated mutagenesis in driving aging phenotypes. Variability among study design and techniques used to assess somatic mtDNA mutation has made initially strong conclusions more nuanced and, in some cases, has led to apparent conflicting results. The major differences among studies related to the type of sample, the technique chosen to detect somatic mutation, and the analysis have all played a part in adding to the confusion. More rigorous study designs looking at not just lifespan, but also molecular and physiological phenotypes across many tissue and cell types, will be important in more firmly establishing a role of mtDNA mutations in aging. In addition, isolating the effects of aging-linked *de novo* mutations versus inherited and early arising mutations, a confounder in many current models, is also an important question in need of attention. Regardless of the role mtDNA mutations play in driving aging directly, understanding their etiology and biology will likely be important in our understanding of why certain age-related diseases, such as AD or PD, frequently exhibit elevated mutations. In addition, the etiology of mutations could provide clues for more direct drivers of aging, as well as inherited disease causing variants.

Some missing aspects in our understanding of the biology of mtDNA mutations will undoubtedly be helped by the use of ever improving technologies. Previously used techniques to detect mtDNA mutations, such as standard or long-range PCR, have been slowly replaced by NGS. NGS has substantially improved the accuracy of mtDNA detection and it is now relatively easy to sequence the entire mtDNA in a population. However, NGS is strongly affected by PCR-induced errors, pseudogene artifacts, *ex vivo* DNA oxidation, and base-calling errors, limiting its ability to detect low level mtDNA mutations, which occur ∼100-fold lower than the error background of these platforms. Error-corrected NGS methods have been developed and are increasingly being used, but conventional NGS is, unfortunately, still the norm. These more modern high accuracy methods have changed our view of the mutagenic processes that act on mtDNA and need to be more frequently used in studies related to somatic mutagenesis.

While an important step forward, most NGS studies are limited by their frequent use of tissue homogenates as a source of DNA, which necessarily removes cell and tissue specific relationships that could provide important information on regarding phenotypic thresholds, clonality, and heteroplasmy that are an important aspect of establishing the impact of potentially pathogenic mtDNA mutations. Laser capture microscopy has partially addressed these issues and has established that clonal expansions of mtDNA mutations are a biological phenomenon. However, the approach is likely too low throughput to deploy at scales high needed to better understand the true burden of clones within a tissue. Embracing emerging single-cell and spatial technologies will allow for a fuller understanding of how mtDNA impact cell and organ function in a more proper biological context. Taken together, it is clear that much remains to be done in order to more fully understand mitochondrial mutagenesis. With the emergence of new capabilities, the future remains bright that a number of long proposed hypotheses related to when, where, and how somatic mtDNA mutations influence aging and disease will soon be answerable.
